# Supplementary material for: Visualising quantum innovation: A regional case study
Source: PLoS One. 2024 Jun 24;19(6):e0305140. doi: 10.1371/journal.pone.0305140 (PMC11195939; doi:10.1371/journal.pone.0305140)
Supplement: S1 Table — (DOCX) [file pone.0305140.s001.docx]

**S1 Table. Literature Review breakdown**

| **AUTHOR** | **OBJECTIVE** | **TYPE OF PUBLICATION** | **METHODO-LOGY** | **KEY FINDINGS** | **GAPS IN RESEARCH** |
| --- | --- | --- | --- | --- | --- |
| Garcia A, et al.    (2023) | -Inform companies about  the state of quantum technologies.    -Offer recommendations to incorporate them in their businesses.    -Listen to their expectations and establish the basis for a national strategy. | -Business report. | -Mapping out every actor in the Spanish ecosystem through own sources.    -Interviewing sector leaders to establish their actions and expectations about quantum. | - Small ecosystem with low visibility.    -Room for improvement in cooperation between actors.    -Talent gap.    -Public investment and coordination are paramount. | -Lack of external sources for named actors in the ecosystem.    -Lack of external viewpoints (strategy is determined by leading companies). |
| Mohr N, et al.    (2022) | -Provide an overview of the global quantum technology space and the industry’s maturity towards them. | -Business report. | -Mapping out the investment in startups extracted from source PìtchBook.    -Calculating market sizes, number of players and other indicators though own research. | -Funding surpassed $1.4 billion, with quantum computing receiving the most.    -Startup creation has slowed.    -Finance and life sectors will see the highest impact.    -US leads the market but China is catching up.    -Serious talent gap. | -Not focused on the Spanish case.    -Lack of detail.    -Output oriented. |
| Chow J, et al.    (2022) | -Inform about the state of quantum computing. | -Business report. | -Mapping out investment numbers from several sources.    -Analyzing the technology through internal council. | -Quantum computing receives funding from most G20 countries but needs further action.    -The biggest impact will be seen on materials science and modeling complex systems.    -There is no advantage over quantum computers nor unified approach. | -Not focused on Spanish case.    -Lack of external sourcing in use-cases and state of the technology. |
| Almasque E, et al.    (2022) | -Inform about industry readiness to quantum technologies. | -Business report. | -Surveying 174 business leaders to establish their actions and expectations about quantum. | -63% believe  quantum computing will be commercialized in 5 years.    -91% are funding quantum R&D or planning to do so.    -Software development is a priority.    -Talent gap is causing deceleration. | -Numbers in the Spanish case can be different.    -Relies entirely on one type of source.    -Expectations do not necessarily correlate with reality. |
| Van Velzen J, et al.    (2022) | -Help businesses identify opportunities for quantum investment and advantage. | -Business report. | -Surveying 200 executives to establish their actions and expectations about quantum.    -Detailed interviewing of 30+ sector leaders. | -23% are working on quantum or planning to.    -Quantum cryptography is already commercialized.    -Quantum computing needs 5-10 years to reach the market. | -Numbers in the Spanish case can be different.    -Relies mostly on inside perspectives. |
| Castelein W, et al.    (2023) | -Map the European quantum technology landscape.    -Define governmental involvement in quantum technologies. | -Governmental report. | -Making an inventory of the 41 members of the QuantERA Consortium. | -Ther has been an increase in national strategies.    -10 countries have national funding programs, but most do not have a centralized funding system. | -Lack of detail.    -Very policy-oriented.    -Very sparce on the Spanish case. |
| Hughes C, et al.    (2022) | -Define the jobs, skills and types of degrees needed for the quantum workforce. | -Academic article. | -Surveying 57 QED-C member companies. | -Both highly specialized and general jobs are needed.    -These broader jobs do not require specific skills.    -A range of degree levels are needed. | -Only focused on jobs.    -Does not detail the nature or location of the companies surveyed. |
| Seskir ZC, et al.    (2022) | -Mapping and categorizing quantum technologies startups. | -Academic article. | -Compiling dataset through different internet sources (442 companies). | -More than 90% have been founded in the last 10 years.    -Most are located in US, UK or Canada. | -Only focused on startup numbers.    -Not focused on the Spanish case.    -Focused only on the private sector. |
| Roberson T.    (2021) | -Applying the innovation ecosystem theory to illustrate the relations between quantum actors. | -Academic article. | -Analyzing granted patents in the US. | -Uptick in number of patents on the last 3 years.    -There are four main technological clusters.    -Academia plays a relevant role in knowledge creation. | -Only illustrates one output of the actors.    -Does not mention the Spanish case.    -Focused only on the private sector. |
