# Supplementary material for: Visualising quantum innovation: A regional case study
Source: PLoS One. 2024 Jun 24;19(6):e0305140. doi: 10.1371/journal.pone.0305140 (PMC11195939; doi:10.1371/journal.pone.0305140)
Supplement: S2 Table — (DOCX) [file pone.0305140.s002.docx]

**S2 Table. Actor breakdown**

| **NAME** | **SOURCE** | **FUNDING ROLE** | **TYPE OF ACTOR** | **Q-FIELD** |
| --- | --- | --- | --- | --- |
| European Comission | CORDIS | Provider | Government | All Fields |
| KPMG | OWN WEBPAGE | NS | Large Company | All Fields |
| Qurv | AMETIC | Receiver | Startup | Sensing |
| CTTC | AMETIC | Receiver | Independent Research Centre | Communications |
| Consell de Tecnologies Quàntiques de Catalunya | AMETIC | NS | Government | All Fields |
| ICFO | AMETIC | Receiver | Independent Research Centre | All Fields except Chemistry |
| LuxQuanta | AMETIC | Receiver | Startup | Cryptography and Communications |
| QuSide | AMETIC | Receiver | Startup | Cryptography |
| BSC | AMETIC | Receiver | Independent Research Centre | Computing |
| ICN2 | AMETIC | Receiver | Independent Research Centre | Computing and Cryptography |
| The Institute of Microelectronics of Barcelona | AMETIC | NS | Independent Research Centre | Computing |
| IFAE | AMETIC | Receiver | Independent Research Centre | Computing |
| IESE Bussiness School | GENERALITAT | Receiver | University Research Centre | All Fields |
| UAB | CORDIS | Receiver | University Research Centre | Computing, Communications, Sensing and Simulation |
| UB | CORDIS | Receiver | University Research Centre | Computing |
| UPC | CORDIS | Receiver | University Research Centre | Computing, Communications, Sensing and Simulation |
| The Quantum Information and CyberseQurity Think Tank | AMETIC | NS | Hub | All Fields |
| Barcelona Institute of Science and Technology | OWN WEBPAGE | NS | Independent Research Centre | All Fields |
| i2CAT | AMETIC | Receiver | Independent Research Centre | Communications |
| Cellnex Telecom | OWN WEBPAGE | Receiver | Independent Research Centre | Communications |
| Grupo AIA Aplicaciones en Informática Avanzada | OWN WEBPAGE | NS | Independent Research Centre | Computing |
| GTD | OWN WEBPAGE | Provider | SME | Computing |
| Sateliot | CORDIS | Receiver | SME | Cryptography and Communications |
| Zymvol Biomodeling SL | OWN WEBPAGE | Receiver | SME | Chemistry |
| Pharmacelera | CORDIS | Receiver | SME | Chemistry |
| Keysight Technologies | AMETIC | NS | Large Company | Computing, Communications and Sensing |
| Centres de Recerca de Cataluya | GENERALITAT | NS | Hub | All Fields |
| Generalitat de Catalunya | GENERALITAT | Provider | Government | All Fields except Chemistry |
| Repsol | OWN WEBPAGE | Receiver | Large Company | Computing |
| Microsoft | AMETIC | NS | Large Company | Computing |
| Consorci de Serveis Universitaris de Catalunya | OWN WEBPAGE | Receiver | Hub | Computing |
| Leitat Technological Center | GENERALITAT | Receiver | Independent Research Centre | Computing |
| Eurecat | AMETIC | Receiver | Independent Research Centre | Computing |
| Everis NTT DATA | OWN WEBPAGE | NS | SME | Computing |
| GMV Innovating Solutions | AMETIC | Receiver | SME | Computing |
| CSIC | AMETIC | Both | Government | All Fields except Chemistry |
| Geonumerics SL | EDF | Receiver | SME | Sensing |
| IQUA Robotics SL | EDF | Receiver | SME | Sensing |
| secpho | OWN WEBPAGE | NS | SME | Computing |
| Banco Santander | NEWS SOURCE | NS | Bank | Cryptography |
| BBVA | OWN WEBPAGE | Receiver | Bank | Computing |
| ICREA | OWN WEBPAGE | NS | Independent Research Centre | All Fields |
| 6G-OPENSEC-KEYS | SPANISH GOVERNMENT | Both | Project | Cryptography |
| IBM | AMETIC | Receiver | Large Company | Computing |
| QUANTUMGRAIN | CORDIS | Both | Project | Simulation |
| ERIDIAN | CORDIS | Both | Project | Computing and Cryptography |
| Imaging-XChem | CORDIS | Both | Project | Chemistry |
| Tecnobit SL | SPANISH GOVERNMENT | Receiver | SME | Communications |
| DECRESIM | CORDIS | Both | Project | Computing and Cryptography |
| NANOCOMP | CORDIS | Both | Project | Computing |
| NOQIA | CORDIS | Both | Project | Computing |
| TOPOQDot | CORDIS | Both | Project | Computing |
| NEWSPIN | CORDIS | Both | Project | Computing |
| CERQUTE | CORDIS | Both | Project | Computing |
| Non-Specified Sources | CORDIS | Provider | Government | Sensing |
| AVaQus | CORDIS | Both | Project | Computing |
| TOCHA | CORDIS | Both | Project | Communications |
| 2D-SIPC | CORDIS | Both | Project | Computing |
| OPTOlogic | CORDIS | Both | Project | Computing |
| Deloitte | AMETIC | NS | Large Company | Computing |
| Shannon | OWN WEBPAGE | Both | Project | Computing |
| Square | CORDIS | Both | Project | Computing |
| Memory | OWN WEBPAGE | Both | Project | Communications |
| CV-QKD | OWN WEBPAGE | Both | Project | Communications |
| QUPIC | CORDIS | Both | Project | Computing and Cryptography |
| PharmScreen2 | CORDIS | Both | Project | Chemistry |
| Estimation Q Sensing | OWN WEBPAGE | Both | Project | Sensing |
| QUANTUM SENSOR NMR | OWN WEBPAGE | Both | Project | Sensing |
| OPENQPU | OWN WEBPAGE | Both | Project | Computing |
| QuasiBohm | OWN WEBPAGE | Both | Project | Computing |
| QUASI-CAT | OWN WEBPAGE | Both | Project | Computing |
| CUCO | AMETIC | Both | Project | Computing |
| PERTE VEC | SPANISH GOVERNMENT | Both | Project | Computing |
| Sener SA | SPANISH GOVERNMENT | Receiver | SME | Communications |
| QUANGO | AMETIC | Both | Project | Cryptography and Communications |
| Q-SiNG | AMETIC | Both | Project | Sensing |
| Quantum Spain | AMETIC | Both | Project | Computing |
| Qrange | CORDIS | Both | Project | Communications |
| CiViQ | CORDIS | Both | Project | Communications |
| DIH4CAT | GENERALITAT | Both | Hub | All Fields |
| European Quantum Flagship | CORDIS | Both | Project | All Fields except Chemistry |
| QuantERA | CORDIS | Both | Project | All Fields |
| Agencia Estatal de Investigación | CORDIS | Receiver | Government | All Fields |
| Gobierno de España | SPANISH GOVERNMENT | Provider | Government | All Fields |
| UAM | CORDIS | Receiver | University Research Centre | Computing and Communications |
| Universitat de València | CORDIS | Receiver | University Research Centre | Computing, Cryptography, Communications and Sensing |
| Universidad de Santiago de Compostela | CORDIS | Receiver | University Research Centre | Computing and Communications |
| Simune Atomistics SL | CORDIS | Receiver | SME | Computing |
| Multiverse Computing | AMETIC | Receiver | Startup | Computing |
| Hispasat S.A. | SPANISH GOVERNMENT | Receiver | SME | Communications |

| QNetworks | OWN WEBPAGE | Both | Project | Cryptography and Communications |
| --- | --- | --- | --- | --- |
| CESGA | AMETIC | Receiver | Independent Research Centre | Computing |
| Centro de Supercomputación de Castilla y León | OWN WEBPAGE | Receiver | Independent Research Centre | Computing |
| Universidad de Zaragoza | OWN WEBPAGE | Receiver | University Research Centre | Computing |
| Fundación Computación y Tecnologías Avanzadas de Extremadura | OWN WEBPAGE | Receiver | Independent Research Centre | Computing |
| Instituto de Astrofísica de Canarias | OWN WEBPAGE | Receiver | Independent Research Centre | Computing |
| Centro de Investigaciones Energéticas Medioambientales y Tecnológicas | OWN WEBPAGE | Receiver | Independent Research Centre | Computing |
| Navarra de Servicios y Tecnologías | OWN WEBPAGE | Receiver | SME | Computing |
| Universidad de Málaga | OWN WEBPAGE | Receiver | University Research Centre | Computing |
| Universidad de Cantabria | OWN WEBPAGE | Receiver | University Research Centre | Computing |
| Indra Sistemas S.A. | SPANISH GOVERNMENT | Receiver | SME | Communications |
| QuantumCat | AMETIC | Both | Project | Computing, Communications, Sensing and Simulation |
| Das Photonics | AMETIC | Receiver | SME | Computing |
| Alter Technology Tuv Nord SA | SPANISH GOVERNMENT | Receiver | SME | Communications |
| Comunidades Autónomas | SPANISH GOVERNMENT | Provider | Government | Communications |
| European Defense Fund | EDF | Provider | Government | Sensing |
| UPM | CORDIS | Receiver | University Research Centre | Computing, Communications and Sensing |
| Telefonica | AMETIC | Receiver | Large Company | Communications |
| VLC Photonics | AMETIC | Receiver | SME | Communications and Sensing |
| CVC Computer Vision Center | GENERALITAT | Receiver | University Research Centre | Computing |
| Acció | GENERALITAT | Receiver | Hub | All Fields |
| Barcelona City Council | OWN WEBPAGE | Provider | Government | Cryptography, Communications and Sensing |
| Renault | SPANISH GOVERNMENT | Receiver | Large Company | Computing |
| Qucats | CORDIS | Both | Project | All Fields |
| QSNP | CORDIS | Both | Project | Cryptography and Communications |
| Universidad de Vigo | CORDIS | Receiver | University Research Centre | Cryptography and Communications |
| S2Quip | CORDIS | Both | Project | Computing, Communications and Sensing |
| Quantum Internet Alliance | CORDIS | Both | Project | Computing |
| Plan Complementario de Comunicación Cuántica | SPANISH GOVERNMENT | Both | Project | Communications |
| AQTION | CORDIS | Both | Project | Computing |
| UCM | CORDIS | Receiver | University Research Centre | Computing |
| OpenSuperQ | CORDIS | Both | Project | Computing |
| Universidad del País Vasco | CORDIS | Receiver | University Research Centre | All Fields |
| Universidad Politécnica de Valencia | CORDIS | Receiver | University Research Centre | Computing |
| Pimec | GENERALITAT | Receiver | SME | All Fields |
| Foment | GENERALITAT | Receiver | Hub | All Fields |
| Castelldefels City Council | OWN WEBPAGE | Provider | Government | Computing, Communications and Sensing |
| CELLEX Foundation | OWN WEBPAGE | Provider | SME | Cryptography |
| laCaixa Foundation | OWN WEBPAGE | Provider | Bank | Cryptography and Communications |
| PASQuanS | CORDIS | Both | Project | Simulation |
| Accenture | AMETIC | Receiver | Large Company | Computing, Cryptography and Communications |
| Amazon Bracket | AMETIC | NS | Large Company | Computing |
| ATOS | AMETIC | NS | Large Company | Computing and Cryptography |
| Grant Thornton | AMETIC | NS | Large Company | All Fields |
| Arquimea Research Center | AMETIC | Receiver | Independent Research Centre | Computing, Cryptography, Communications and Sensing |
| QCIRCLE | CORDIS | Both | Project | Computing, Cryptography, Communications and Sensing |
| aQuantum software engineering | AMETIC | NS | Startup | Computing |
| Cinfo | AMETIC | NS | Large Company | Computing |
| Entanglement Partners SL | AMETIC | NS | Startup | All Fields |
| G2-Zero | AMETIC | Receiver | Startup | Communications |
| Inspiration-Q | AMETIC | Receiver | Startup | Computing |
| IPronics | AMETIC | Receiver | Startup | Computing |
| IQM | AMETIC | Receiver | Large Company | Computing |
| Qcentroid | AMETIC | NS | Startup | Computing |
| Quantum Mads | AMETIC | NS | Startup | Computing |
| Quamvia | AMETIC | NS | Startup | Computing |
| Serikat Servicios Informáticos | AMETIC | NS | Large Company | Computing |
| TTI Norte | AMETIC | Receiver | SME | Computing and Communications |
| QMiCS | CORDIS | Both | Project | Computing and Communications |
| NEASQC | CORDIS | Both | Project | Computing |
| EY | AMETIC | NS | Large Company | Computing |
| Ibermática | AMETIC | Receiver | Large Company | Computing |
| Universidad da Coruña | OWN WEBPAGE | Receiver | University Research Centre | Computing and Communications |
| CTIC | AMETIC | Receiver | Hub | Computing |
| DIPC | AMETIC | Receiver | Independent Research Centre | All Fields |
| Gobierno Vasco | OWN WEBPAGE | Provider | Government | All Fields |
| Diputación Foral de Gipuzkoa | OWN WEBPAGE | Provider | Government | All Fields |
| Kutxa Fundazioa | OWN WEBPAGE | Provider | Bank | All Fields |
| Fundación EDP | OWN WEBPAGE | Provider | Large Company | All Fields |
| Prometheus | CORDIS | Both | Project | Computing |
| CAF | OWN WEBPAGE | Provider | Large Company | All Fields |
| Ayuntamiento de San Sebastián | OWN WEBPAGE | Provider | Government | All Fields |
| ColQDMol | SPANISH GOVERNMENT | Both | Project | Sensing |
| Fundación IMDEA | AMETIC | Receiver | Independent Research Centre | Communications |
| Tecnalia | AMETIC | Receiver | Independent Research Centre | All Fields |
| AMETIC | AMETIC | Receiver | SME | All Fields |
| Disruptive | AMETIC | Receiver | Hub | Computing |
| Caramuel | SPANISH GOVERNMENT | Both | Project | Cryptography and Communications |
| Gipuzkoa Quantum | AMETIC | NS | Hub | All Fields |
| IKUR | AMETIC | NS | Hub | All Fields |
| Madrid Quantum | AMETIC | Both | Project | Communications |
| Instituto Nacional De Técnica Aeroespacial | SPANISH GOVERNMENT | Receiver | Independent Research Centre | Communications |
| Fundación Vithas | OWN WEBPAGE | Receiver | Independent Research Centre | Communications |
| Centro Español de Metrología | OWN WEBPAGE | Receiver | Government | Communications |
| Polo de Tecnologías Cuánticas de Galicia | AMETIC | Both | Hub | Computing and Communications |
| Xunta de Galícia | OWN WEBPAGE | Provider | Government | Computing and Communications |
| Quantica | AMETIC | NS | Hub | All Fields |
| QSpain | AMETIC | NS | Project | Computing |
| Bizkaia Quantum Ecosystem | AMETIC | Both | Hub | All Fields |
| Diputación Floral de Bizkaia | NEWS SOURCE | Provider | Government | All Fields |
| Ayuntamiento de Bilbao | NEWS SOURCE | Provider | Government | All Fields |
| GAIA | NEWS SOURCE | Receiver | Hub | All Fields |
| Silicon Europe | NEWS SOURCE | Receiver | Hub | All Fields |
| Universidad de Deusto | NEWS SOURCE | Receiver | University Research Centre | All Fields |
| Mondragon Unibertsitatea | NEWS SOURCE | Receiver | University Research Centre | All Fields |
| Quantum World Association | AMETIC | NS | Hub | Computing |
| Quantum Information Network in Spain | AMETIC | Both | Hub | All Fields |
| Universidad de Sevilla | OWN WEBPAGE | Receiver | University Research Centre | All Fields |
| Universidad de Granada | OWN WEBPAGE | Receiver | University Research Centre | All Fields |
| Universidad de Zaragoza | OWN WEBPAGE | Receiver | University Research Centre | All Fields |
| AgrarIA | AMETIC | Both | Project | Computing |
| 1A INGENIEROS S.L.P. | SPANISH GOVERNMENT | Receiver | SME | Computing |
| CODESIAN SOFTWARE TECH S.L. | SPANISH GOVERNMENT | Receiver | SME | Computing |
| CASA AMETLLER | SPANISH GOVERNMENT | Receiver | SME | Computing |
| Celtiberian Solutions S.L. | SPANISH GOVERNMENT | Receiver | SME | Computing |
| DRONETOOLS S.L. | SPANISH GOVERNMENT | Receiver | SME | Computing |
| EMERGYA INGENIERIA SL | SPANISH GOVERNMENT | Receiver | SME | Computing |
| FLORETTE IBÉRICA S.L. | SPANISH GOVERNMENT | Receiver | SME | Computing |
| LANZADERA DIGITAL SL | SPANISH GOVERNMENT | Receiver | SME | Computing |
| GRUPO HISPATEC INFORMÁTICA EMPRESARIAL S.A | SPANISH GOVERNMENT | Receiver | SME | Computing |

| Inteligencia Solagua S.L. | SPANISH GOVERNMENT | Receiver | SME | Computing |
| --- | --- | --- | --- | --- |
| Instituto Tecnológico de Castilla y León | SPANISH GOVERNMENT | Receiver | Independent Research Centre | Computing |
| AGROINDUSTRIAL KIMITEC S.L. | SPANISH GOVERNMENT | Receiver | SME | Computing |
| KIVNON LOGISTICA SL | SPANISH GOVERNMENT | Receiver | SME | Computing |
| LB-BAGGING S.L. | SPANISH GOVERNMENT | Receiver | SME | Computing |
| PRIMAFRIO SL | SPANISH GOVERNMENT | Receiver | SME | Computing |
| Secmotic Innovation S.L. | SPANISH GOVERNMENT | Receiver | SME | Computing |
| Sylentis S.A | SPANISH GOVERNMENT | Receiver | SME | Computing |
| TEPRO Consultores Agrícolas SL | SPANISH GOVERNMENT | Receiver | SME | Computing |
| MIGUEL TORRES S.A. | SPANISH GOVERNMENT | Receiver | SME | Computing |
| Universidad de Salamanca | SPANISH GOVERNMENT | Receiver | SME | Computing |
| QFirst | AMETIC | Both | Project | Sensing |
| TEKNIKER | OWN WEBPAGE | Receiver | Independent Research Centre | Sensing |
| Materials Physics Center | OWN WEBPAGE | Receiver | Independent Research Centre | Sensing |
| AVS NEXT | OWN WEBPAGE | Receiver | SME | Sensing |
| Quantek | AMETIC | Both | Project | Computing |
| QuPilot | OWN WEBPAGE | Both | Project | Computing, Communications and Sensing |
| Gobierno de Asturias | OWN WEBPAGE | Provider | Government | Computing |
| European Space Agency | SPANISH GOVERNMENT | Provider | Government | Communications |
| Thales Alenia Space España SA | SPANISH GOVERNMENT | Receiver | SME | Communications |
