# Supplementary material for: Visualising quantum innovation: A regional case study
Source: PLoS One. 2024 Jun 24;19(6):e0305140. doi: 10.1371/journal.pone.0305140 (PMC11195939; doi:10.1371/journal.pone.0305140)
Supplement: Attachment — Submitted filename: Response to Reviewers.docx [file pone.0305140.s003.docx]

**Response to the reviewers**

Dear Editor

We are very happy to know that our paper “Visualizing Quantum Innovation: A regional Case Study” meets the merits for publication in PLOS One while requiring minor revisions.

In what follows we, present a point-by-point response to the editor and reviewer’s comments and suggestions as well an important modification to the authors list that that has been decided internally.

**Academic editor**: “The introduction provides a good, generalized background of the topic that quickly gives the reader an appreciation of the wide range of applications for this technology.”

**Authors**: We would like to thank the editor for appreciating this value of our article

**Academic editor**: “However, to make the motivation clearer and to differentiate the paper some more from other applied papers, the author may wish to provide another sentence giving examples of some of the applications of this technology, along with appropriate references.”

**Authors**: We have added 8 new citations including those that the editor kindly recommend in the introductory sentence: “*Potential application of quantum technology range drug discovery (Santagati et al, 2024) , financial modelling (Herman et al, 2023) , weather forecasting (Tennie et al, 2023), cybersecurity (Neil, 2023) , machine learning algorithms (Biamonte et al, 2017, Khan et al, 2022) and Bloch chain (Wang et al, 2022, Chinnasamy et al, 2023)*”

**Academic editor**: The objective is clearly defined in the last paragraph of introduction section and clearly mentions the flow of the paper. The experimental apparatus is quite standard, and is appropriate for the study, especially given that the focus of the paper is to develop a privacy preserving platform for industrial applications.

**Authors**: We thank the editor

**Reviewer #1**: The article is based on country specific. The authors present a literature review that considers a set of reports by several organizations.
Author should mention the name of organization too.
All the tables and figures should be described within the article.

**Authors**: We have included the name of the organizations in the section “Structure of the Study” in the sentence: “*we present a literature review that considers a set of reports by several organizations, like the World Economic Forum, Ametic, McKinsey, Quantum Flagship, IBM, IQM, among others*,”

**Reviewer #2**: this research has collected comprehensive data and conducted extensive analysis regarding the quantum technological innovation Spain. the results of this research may be expanded to other regions in Europe, as well as help in the potential development of quantum technologies globally.

**Authors**: We thank reviewer #2 for her/his encouraging comment.

Yours sincerely,

Osvaldo Jiménez Farías and Eóin Phillips

On behalf of the authors.
